# The modulation of mRNA levels of MAOA by electroacupuncture and psychotherapy in patients with pathological internet use

**DOI:** 10.3389/fpsyt.2022.918729

**Published:** 2022-08-11

**Authors:** Yu Dai, Chenchen Zhang, Lingrui Zhang, Chao Wen, Tianmin Zhu

**Affiliations:** ^1^Department of Traditional Chinese Medicine, Chengdu Eighth People’s Hospital (Geriatric Hospital of Chengdu Medical College), Chengdu, China; ^2^College of Rehabilitation and Health Preservation, Chengdu University of Traditional Chinese Medicine, Chengdu, China; ^3^Department of Rehabilitation, Traditional Chinese Medicine Hospital of Longquanyi District, Chengdu, China; ^4^Department of Medicine, Leshan Vocational and Technical College, Leshan, China

**Keywords:** electroacupuncture, psychotherapy, pathological internet use, MAOA, mRNA level

## Abstract

**Objective:**

The aim of this study was to observe the efficacy of electroacupuncture (EA) and psychotherapy (PT) effect on the mental status, sleep quality and impulsive trait in patients with pathological internet use, and to observe the changes of Monoamine oxidase type A (MAOA) messenger Ribonucleic acid (mRNA) levels in each group.

**Methods:**

A total of 60 PIU patients were included for the present study. These patients were randomly divided into two groups: EA group and PT group. Baihui, Sishencong, Hegu, Neiguan, Shenmen, Taichong, Sanyinjiao and Xuanzhong were selected for acupuncture in the EA group, while group psychotherapy combined with individual psychotherapy was used for intervention in patients in the PT group. Young’s Internet addiction Test (IAT), Yale-Brown Obsessive-Compulsive Scale (Y-BOCS), Self-Rating Anxiety Scale (SAS), Self-Rating Depression Scale (SDS), Barratt Impulse Scale (BIS-11) and Pittsburgh Sleep Quality Index (PSQI) were used to observe the severity of Internet addiction, mental status, sleep quality and impulsive trait of all patients at baseline and 40th days of treatment; and MAOA mRNA data were collected at baseline and 40th days of treatment.

**Results:**

Electroacupuncture and psychological intervention effectively reduced IAT, SAS, SDS, Y-BOCS, BIS and PSQI scores of PIU patients. After 40 days treatment, the MAOA expression of the PT group was increased, and there was no significant change in EA group. The correlation analysis indicated that IAT scores were positively correlated with SAS, SDS, Y-BOCS, BIS and PSQI at baseline. In addition, after treatment the EA group showed that the change in IAT scores was positively correlated with the change in Y-BOCS and BIS scores, and the PT group showed that the change in IAT scores was positively correlated with the change in SDS, BIS and PSQI scores.

**Conclusion:**

The present study showed that electroacupuncture and psychological intervention can improve severity of Internet addiction, mental status, sleep quality and impulsive trait of PIU patients. Simultaneously, neurobiological changes may be the underlying mechanisms of psychotherapy for internet additcion.

## Introduction

In the past decades, with the rapid development of computer and Internet technology, the Internet has changed the way we communicate, exchange information, and participate in real-time events thousands of miles away. It has become an indispensable part of people’s life, work and entertainment. Although the Internet has brought more convenience to our lives, the improper use of the Internet may cause serious damage to society, work and personal psychology (1–3). This brings an emerging problem — pathological internet use (PIU) or internet addiction disorder (IAD), which is a behavioral addiction (4). This can be defined as “creating mental, social, school and/or work difficulties in one’s life by using the Internet” (5). Some research have revealed that the incidence of PIU is increasing, and tends to involve younger subjects (6–8), leading to a series of family and social problems. Therefore, PIU has received increasing attention, and is gradually being recognized in the field of public health.

At present, the etiopathogenisis and pathogenesis for PIU have not been elucidated, and its treatment remains in the exploratory stage. The treatments for PIU at home and abroad are mainly the following: psychotherapy, drug therapy or the combination of both. These interventions have been proven to reduce the time for using the internet, and improve the psychological state of Internet addicts (9, 10). Although these treatments have curative effects, they also have some side effects, especially drug therapy (11, 12). If Internet addicts administer related therapeutic drugs for a long period of time, this may lead to serious adverse reactions. Therefore, some researchers have used traditional Chinese medicine to intervene in PIU, and our previous studies also showed that electroacupuncture can improve the clinical symptoms of Internet addicts (13, 14). More and more studies used fMRI to research the mechanism of acupuncture because for fMRI can visualize the effects of acupuncture (15, 16). Previous studies have confirmed that acupuncture has a regulatory effect on the structure and function of brain regions in substance addicts (17, 18). Similarly, our previous findings suggest that acupuncture on PIU individuals can regulate functional connectivity of reward and habit systems (19), and this result is related to the current research results of Internet addiction (20). In a review study, Weinstein and Lejoyyeux (21) reported that Internet addiction is associated with the brain regions associated with reward and cognitive control network. Liu et al. (22) also suggested that the functional change of brain in PIU patients may be relative to reward pathways. Therefore, the modulation effect of acupuncture on brain regions in PIU individuals, which might be the underlying mechanisms of acupuncture on PIU. A large number of studies revealed that psychotherapy can reduce the online time, negative mood, compulsive behavior and so on, but these studies only by using relevant scales to explore the effect of psychotherapy on PIU individuals (23–25).

Dopaminergic and serotoninergic systems are closely related to reward pathway. One study showed that dopamine (DA) played an important role in PIU (26). Hou et al. (27) reported that the individuals with PIU had a decreased level of expression of dopamine transporter in the striatum compared to controls. Luo et al. (28) study indicated that serotonin (5-HT) level was related with PIU. Furthermore, MAOA plays a crucial role in the metabolism of 5-HT, DA and norepinephrine (NE) (29). Low MAOA activity may raise the levels of 5-HT and DA that produce abnormal neurotransmitter system development and behavior (30). Recent research proved that high-activity of the MAOA gene can make the rapid catalyzation of 5-HT and NE, thereby leading to depression (31). Juanes et al found that after long-term drinking, the rhesus macaque MAOA expression in blood decreased and the level of dopamine in cerebrospinal fluid increased (32). Therefore, we speculated that there may be a mechanistic link between MAOA gene and PIU, and our previous research confirmed this hypothesis (33).

Although acupuncture and psychotherapy can improve the symptoms of PIU patients, it is not clear whether acupuncture or psychotherapy can affect the levels of MAOA with PIU patients. In order to provide a new perspective for the study of therapeutic mechanism of PIU, we had made current study. This study aimed to assess (a) the association between MAOA levels of mRNA and therapy; (b) the difference between EA and PT; (c) whether there was an association between changes in MAOA expression in PIU patients and clinical indicators.

## Materials and methods

### Participants

The aim of the study was to evaluate the impact of different treatment methods on PIU patients, so we choose difference between two dependent means (matched pairs) as statistical test, an effect size of *d* = 0.6 with power = 0.85 (α = 0.05; two-tailed) to calculate the sample size by Gpower. G-Power calculated that 27 sample sizes were needed for each group, finally each group recruited 30 participants, considering the dropout rate. Participants were recruited in this study came from University of Electronic Science and Technology, Chengdu University of Traditional Chinese Medicine and Sichuan Vocational and Technical College of Communication. All participants in our study were native Chinese speakers. Patients were diagnosed as PIU based on the Beard’s Diagnostic Questionnaire (5). The inclusion criteria were as follows: (1) conformed to diagnostic criteria; (2) aged between 18 and 30 years; (3) right-handed; (4) fMRI examination without contraindications; (5) have signed informed consent. The exclusion criteria include the following: (1) having undergone any form of therapeutic intervention; (2) having any other organic or mental illnesses; (3) having a history of drug addiction or alcohol; (4) acupuncture treatment allergy; (5) pregnant or breast-feeding women; (6) left-handed. This study underwent ethical scrutiny and was approved by the Ethics Review Board of the Affiliated Hospital of Chengdu University of Traditional Chinese Medicine (Permission number: 2016KL-005) and it has been registered on Chinese Clinical Trial Registry: the registration number is ChiCTR-INR-16008102.

In this study, eligible subjects were numbered by a researcher who was not involved in the experimental process and assigned to two groups using a randomized digital table produced by SAS 8.0 software. In order to better implement the blind method, we did not inform participants of the specific content of the treatment at the time of recruitment. Only when participants were confirmed to join a group, they can be told which treatment they would receive. In addition, participants in each group were unaware of the other groups. When performing analysis, statisticians were not informed about the group allocation.

### Questionnaire

The following scales were used to examine the subjects’ the severity of pathological internet use, anxiety and depression symptoms, compulsiveness, impulsiveness and sleep condition.

Young’s Internet Addiction Test (IAT) (34) is a self-report questionnaire which consists of 20 items to measures the degree of pathological internet use. Participants use this 5-point scale ranging from 1 (never) to 5 (always) to report the frequency with which they engaged in listed Internet behaviors during the past year. The possible score varies from 20 to 100. Several studies have proved that Young’s internet addiction test have adequate validity and reliability (35, 36).

Self-Rating Anxiety Scale (SAS) (37) is widely used to measure participants’s anxiety state. It consists of 20 items, which are scored on a 4-point Likert scale. The total score was calculated by the raw score multiplied by 1.25. Because the Chinese version of the SAS demonstrates adequate reliability and validity in past studies, so the Chinese version of the questionnaire is used in the present study (38, 39).

Self-Rating Depression Scale (SDS) is used to ascertain the individuals’s situations about depression which compiled by William Zung (40). The SDS questionnaire has 20 self-report questions, which are rated on a 4-point scale. The total score was calculated by the raw score multiplied by 1.25. The Chinese version of the SDS has been widely used, and the reliability and validity has been verified in previous studies (41, 42).

Yale-Brown Obsessive-Compulsive Scale (Y-BOCS) is a self-report measure that consists of 10 core items to test the severity of obsessive-compulsive symptoms (43). Each item with five response categories, rating from 0 (no symptoms) to 4 (severe symptoms). The Chinese version of Y-BOCS has been found to have adequate reliability in Chinese samples study, with Cronbach’s alpha *a* = 0.83 (44).

Barratt Impulsiveness Scale (BIS-11) is a self-report questionnaire consisting of 30 items, which are rated on a 4-point scale (1 = rarely/never; 4 = almost always/always), evaluate the impulsivity of individuals (45). This scale includes three impulsiveness subscales: motor, non-planning, and attentional impulsiveness. The Chinese version of the BIS-11 was verified previously (46).

Pittsburgh Sleep Quality Index (PSQI) is the most commonly used instrument to assess the subjective sleep quality of individuals in clinical (47). This scale consists of 19 items measuring 7 components of sleep, including subjective sleep quality, sleep onset latency, total sleep duration, sleep efficiency, sleep disturbances, use of sleep medication, and daytime dysfunction (48). Each component is scored from 0 to 3 points, and each component are summed to get a total score (range 0–21). The Chinese version of the PSQI was verified previously (49).

The patient need a quiet environment to completed clinical assessments. At the same time, the participants needed to remain awake and attentive and follow the professional’s instructions.

### Intervention program

**FIGURE 1:**
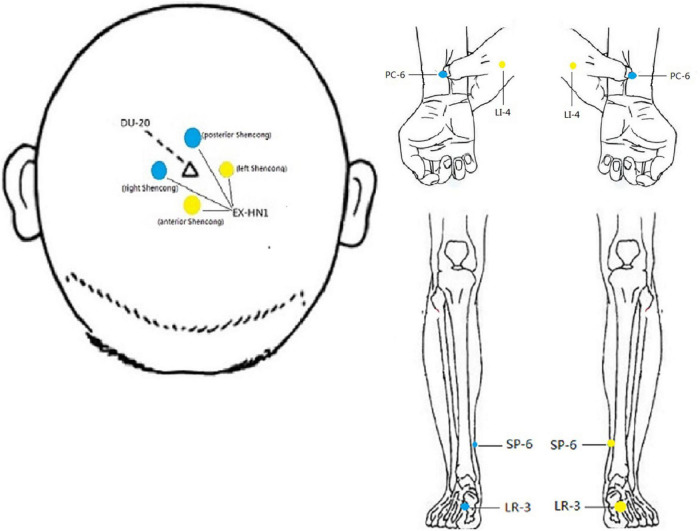
Location of acupoints receiving stimulation. Yellow represents the left, Blue represents the right. The left and right acupoints used alternately, that means stimulating the left acupoints for this time and the right acupoints for next time.

Electroacupuncture Treatment: Hwato brand disposable acupuncture needles (size 0.30 × 40 mm, 0.30 × 25 mm) and G6805 type multichannel electroacupuncture apparatuses were used. According to our previous studies on acupuncture for PIU (13, 14), participants received acupuncture at Baihui (DU-20, located in the center of the top), Sishencong (EX-HN1, a group of four points at the vertex, 1.0 *cun* anterior, posterior and lateral to Baihui, respectively), and bilateral Hegu (LI-4, located between the first and second metacarpal bone, approximately in the midpoint of the second metacarpal radial side), Neiguan (PC-6, located 2 *cun* above the transverse crease of the wrist, between palmaris longus and flexor carpi radialis tendons), Shenmen (HT-7, located ulnar side of the transverse crease of the wrist, in radial depression of the flexor carpi ulnaris tendon), Taichong (LR-3, located in the depression anterior to the meeting point of the first and second metatarsals), Sanyinjiao (SP-6, located 3 *cun* directly above the tip of the medial malleolus, on the posterior border of the medial aspect of the tibia) and Xuanzhong (GB-39, located 3 *cun* above the lateral malleolus, at the anterior edge of the fibula). Acupuncture was performed by an experienced acupuncturist according to the guidelines of acupuncture. Manipulations: After routine disinfection, perpendicularly insert 0.3 mm × 40 mm acupuncture needles, 0.5–1.0 *cun* into LI-4 and PC-6, 1–1.5 *cun* into SP-6 and GB-39, and 0.3–0.5 *cun* into LR-3 and HT-7, and horizontally insert 0.3 mm × 25 mm acupuncture needles, 0.5–0.8 *cun* into DU-20 and EX-HN1. Following needle insertion, uniform reinforcing-reducing manipulations of twirling, lifting, and thrusting were conducted on all needles to reach de qi (De qi refers to the process of the patient produces acupuncture acid, hemp, bilge sensation, and the doctor’s heavy and tight sensation coming from beneath the needle, is considered by acupuncture achieved effect important condition). Three pairs of electrodes from the electric stimulator were connected separately to the needle handles at two points of EX-HN1(the anterior and left Shencong, or the posterior and right Shencong), LI-4 and PC-6, LR-3 and SP-6 (The left and right acupoints used alternately, Figure 1). Stimulus parameters: The frequency of the rarefaction wave was 2 Hz, and the condensation wave was 100 Hz, with a waviness width of 0.3 ms, and the intensity output was gradually adjusted from 0 mA to the extent of the subject’s maximum tolerance. The stimulation lasted for 30 min. Participants received 1 treatment session every other day, 10 sessions as 1 treatment course, 20 sessions in total.

Psychological Treatment: Teamwork and individual counseling were performed by a nationally accredited psychologist. The main steps are as follows: (a) At the first meeting, IAD patients were allowed to be acquainted with each other by participating in a game designed by the psychologist. This would allow these patients to form team consciousness, which is conducive for the subsequent group therapy. The psychologist should understand the past situation of participants, especially their major life events, in order to determine the source of their negative emotions and bad character; (b) During the group psychotherapy, group play therapy was adopted to enhance mutual trust. At the same time, the psychologist should objectively evaluated the internet related events of internet addiction individuals (such as online games, online interpersonal communication, etc.), and compared their past and present situation, in order to let them know that they had deviation cognition on the Internet. During the whole process, the psychologist should allow patients to learn how to actively and correctly deal with problems, and establish a correct behavior pattern; (c) The psychologist formulate a reasonable schedule for patients, according to their own situation, and supplemented this with transference and self-control therapy, thereby lenabling them to determine their positive and beneficial interests. These patients received treatment every 4 days, and each treatment lasted for 2 h. 5 times was considered as one treatment course, and all patients received 2 treatment courses.

### Detection of the relative mRNA expression levels of MAOA gene

The samples of peripheral vein blood were collected from all patients before and after treatment and stored in EDTA anticoagulant tube. Mononuclear cells were isolated using a gradient centrifuge (Thermo, Waltham, MA, United States).

Total RNA Extraction: (1) Whole blood mononuclear cells, about 10^6 were added with 1 ml Trizol, and were repeatedly blown to resuspend; (2) The sample was kept at room temperature for 5 min and fully cracked, and the experiment continued in the following steps; (3) Add 0.2 ml chloroform, mixed for 15 s, and placed at room temperature for 2 min; (4) Centrifuged at 13,000 rpm at 4°C for 15 min, and the upper aqueous phase was absorbed into the EP tube pretreated by DEPC; (5) Add 0.5 ml isopropyl alcohol, reversed and mixed for several times, and precipitate on ice or for 10 min; (6) The supernatant was centrifuged at 13,000 rpm at 4°C for 15 min, and then discarded; (7) Add 1 ml 75% ethanol and mixed it by volute rotation; (8) Centrifuge at 12,000 rpm at 4°C for 5 min, then discard the supernatant. Then use a centrifuge for instantaneous centrifugation, carefully suck up the liquid; (9) Open the lid and dry the RNA for a while, add 20 μl DEPC water to dissolve it, and freeze at -80°C; (10) 1% agarose gel electrophoresis was performed on the total RNA extracted in the end. The RNA integrity was detected using the Agilent 2200 Bioanalyzer (Agilent, CA, United States).

Reverse Transcription: 1 μg of total RNA was reverse-transcribed into 20 μl first-strand cDNA by using the Fermentas cDNA synthesis kit (RevertAidTM, Fermentas, United States) according to the manufacturer’s instruction.

Real-Time PCR: Nucleotide primers for real-time PCR amplification were designed using primer blast software on the National Center for Biotechnology Information website. Primers used for real-time PCR are as follows: β-actin: forward, 5’-GAAGATCAAGATCATTGCTCCT-3’ and reverse, 5’-TTGCTGATCCACA-3’ (amplicon size, 111-bp). MAOA: forward, 5’-CTGCCATCATGGGCTT-3’ and reverse, 5’-TTGCTGATCCACA-3’ (amplicon size, 154-bp). For real-time PCR, the reaction volume was 25 μl/tube (2 × TaqMan Real-time PCR Mix 12.5 μl + PCR primer pair 1.2 μl + fluorescent probe 0.6 μl + ddH2O 7.7 μl and cDNA template 3 μl). The reaction was performed on an FTC-3000QPCR system (Funglyn Biotech, Toronto, Canada). Reaction conditions were as follows: pre-denaturing at 95 °C for 10 min, denaturation at 95 °C for 10 s, annealing at 53 °C for 30 s, and 45 cycles of extension for 30 s at 60 °C. After the reaction, the amplification curves of target gene and internal reference gene were obtained, and the Ct value was calculated. We use the way of Delta-delta Ct to detect the relative mRNA expression in cells.

### Statistical analyses

All data were analyzed by SPSS Statistics 21.0 software and GraphPad Prism 7 Software. If data conforms to the normal distribution, then Independent sample I-test was used for inter group comparison, and Paired sample *t*-test was used for intra group comparison; and if the data follows a non-normal distribution, we used Non-parametric test to compare in this case. While Chi-square test was used for categorical variable (e.g., gender). When doing correlation analysis, the data must be tested for K-S normality. Pearson correlation coefficient was used to analyze the normal distribution, and Spearman correlation coefficient was used to analyze the non-normal distribution. *P* < *0.05* was considered to be statistically significant.

## Results

**FIGURE 2:**
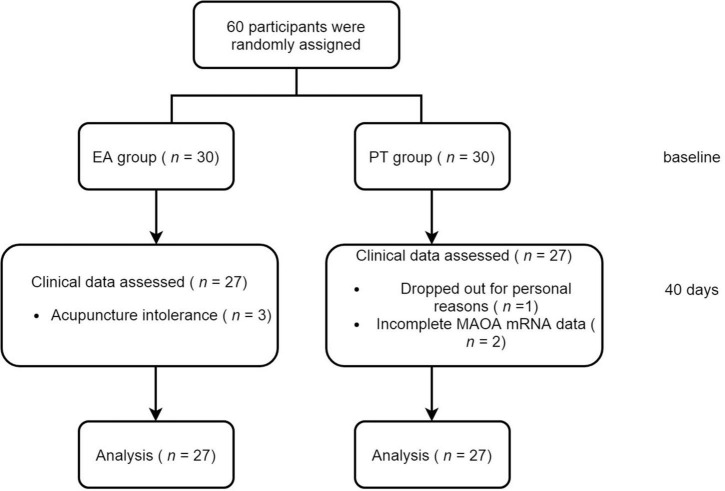
Flow chart.

A total of 60 PIU subjects which were randomly divided into the EA group and the PT group were recruited in our study initially. There were three subjects in the EA group who dropped out due to acupuncture intolerance; and three subjects was excluded in PT group, one for personal reasons and the other two for incomplete MAOA mRNA data. Hence, a total of 54 patients were completed, and the total completion rate was 90%. Flow of participants in the study is shown in Figure 2.

### Demographic characteristics and clinical measures

As shown in Table 1, there were no significant differences in demographics and clinical characteristics data between the EA group and the PT group at baseline (*P* > *0.05*), that means the two groups were comparable for the severity of illness. After 40 days of treatment, the scores of IAT, SDS, SAS, Y-BOCS, BIS-11, and PSQI were decreased in both groups (*P* < 0.05). After 40 days of treatment, there was no significant difference in the scores of IAT, SAS, SDS, Y-BOCS, BIS and PSQI between the two groups (*P* > 0.05).

**TABLE 1:** General characteristics and clinical characteristics of the participants.

	EA group pretreatment (*n* = 27)	EA group posttreatment (*n* = 27)	PT group pretreatment (*n* = 27)	PT group posttreatment (*n* = 27)
Gender, male (*n*,%)	20 (74.07)	/	22(81.48)	/
Age (years)	22.44 ± 2.50	/	21.2 ± 1.78	/
Internet age (years)	7.93 ± 3.09	/	7.88 ± 2.58	/
IAT	65.26 ± 14.63	45.85 ± 12.61*	62.30 ± 11.03	44.26 ± 11.73▲
SAS	48.11 ± 11.15	40.63 ± 8.70*	48.48 ± 10.55	43.11 ± 9.75▲
SDS	55.19 ± 10.90	45.67 ± 7.96*	54.22 ± 9.63	49.33 ± 10.44▲
Y-BOCS	11.33 ± 9.11	6.00 ± 6.59*	12.15 ± 6.38	6.30 ± 5.86▲
BIS-11	75.00 ± 9.35	69.85 ± 9.99*	75.96 ± 9.36	68.19 ± 9.26▲
PSQI	7.92 ± 2.74	5.52 ± 3.07*	8.07 ± 2.84	4.74 ± 2.19▲

IAT, Young’s Internet Addiction Test; SAS, Self Rating Anxiety Scale; SDS, Self Rating Depression Scale; Y-BOCS, Yale-Brown Obsessive-Compulsive Scale; BIS-11, Barratt Impulse Scale; PSQI, Pittsburgh Sleep Quality Index. *Comparison in the EA group between pre- and posttreatment, *P* < 0.05; ▲Comparison in the PT group between pre- and posttreatment, *P* < 0.05.

### MAOA mRNA level results

**FIGURE 3:**
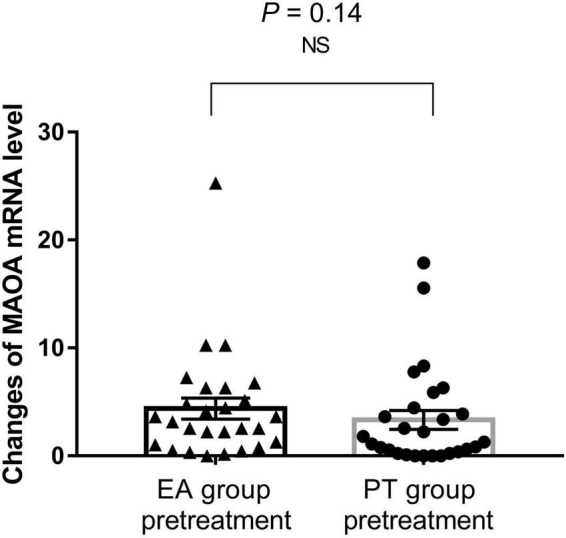
Results of mRNA levels of MAOA in the two groups before treatment. Bars represent means, error bars represent SEM, triangles and circles represent individual data points. NS, indicates not statistically.

Figure 3 shows that there was no significant difference in MAOA expression between patients in the EA group and the PT group at baseline (*P* > *0.05*).

**FIGURE 4:**
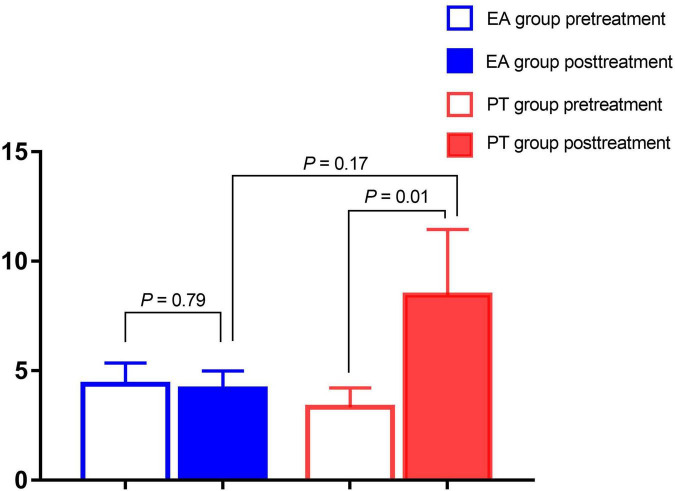
Results of mRNA levels of MAOA pre-and post-treatment.

Figure 4 shows that the MAOA expression of the PT group was increased with that before treatment (*P* < *0.05*). In contrast, the EA group has no significant change (*P* > *0.05*). On the 40th day of treatment, there was no significant difference in the MAOA mRNA levels between the two groups.

### Association between MAOA mRNA levels and clinical scale scores

There was no remarkable association between the relative expression levels of MAOA and IAT, SAS, SDS, Y-BOCS, BIS-11, or PSQI scores in patients with PIU (*P* > 0.05). IAT scores were positively correlated with SAS scores (*r* = 0.36, *P* = 0.007), SDS scores (*r* = 0.292, *P* = 0.032), Y-BOCS scores (*r* = 0.475, *P* = 0.000), BIS scores (*r* = 0.383, *P* = 0.004) and PSQI scores (*r* = 0.354, *P* = 0.009), respectively. (See Table 2).

**TABLE 2:** Correlation between MAOA mRNA levels and clinical measures at baseline.

	MAOA	IAT	SAS	SDS	Y-BOCS	BIS	PSQI
MAOA	1	−0.024	0.003	−0.017	−0.226	0.06	0.139
IAT	−0.024	1	0.36**	0.292*	0.475**	0.383**	0.354**
SAS	0.003	0.36**	1	0.729**	0.34*	0.367**	0.573**
SDS	−0.017	0.292*	0.729**	1	0.309*	0.448**	0.525**
Y-BOCS	−0.226	0.475**	0.34*	0.309*	1	0.254	0.243
BIS-11	0.06	0.383**	0.367**	0.448**	0.254	1	0.397**
PSQI	0.139	0.354**	0.573**	0.525**	0.243	0.397**	1

IAT, Young’s Internet Addiction Test; SAS, Self Rating Anxiety Scale; SDS, Self Rating Depression Scale; Y-BOCS, Yale-Brown Obsessive-Compulsive Scale; BIS-11, Barratt Impulse Scale; PSQI, Pittsburgh Sleep Quality Index.

*P < 0.05; **P < 0.01. Bold values indicate statistical significance.

Table 3 lists the correlations between the changes of expression of MAOA and changes in clinical scores in each group after treatment. The change of MAOA in the EA group was positively correlated with PSQI scores (*r* = 0.428, *P* = 0.026). Table 4 shows the correlations between the changes of IAT scores and changes in other clinical scores in each group after treatment. The chage of IAT scores in the EA group was positively correlated with Y-BOCS (*r* = 0.391, *P* = 0.044), and BIS scores (*r* = 0.501, *P* = 0.008); while the chage of IAT scores in the PT group was positively correlated with SDS scores (*r* = 0.432, *P* = 0.024), BIS scores (*r* = 0.507, *P* = 0.007), and PSQI scores (*r* = 0.444, *P* = 0.02).

**TABLE 3:** Correlation between MAOA change and clinical index change.

	Change in IAT	Change in SAS	Change in SDS	Change in Y-BOCS	Change in BIS	Change in PSQI
EA						
Change in MAOA	0.031	−0.174	−0.235	−0.286	0.233	0.428*
PT						
Change in MAOA	−0.008	0.299	0.31	0.054	0.322	0.143

IAT, Young’s Internet Addiction Test; SAS, Self Rating Anxiety Scale; SDS, Self Rating Depression Scale; Y-BOCS, Yale-Brown Obsessive-Compulsive Scale; BIS-11, Barratt Impulse Scale; PSQI, Pittsburgh Sleep Quality Index.

*P < 0.05. Bold values indicate statistical significance.

**TABLE 4:** Correlation between IAT score change and clinical index change.

	Change in MAOA	Change in SAS	Change in SDS	Change in Y-BOCS	Change in BIS	Change in PSQI
*EA*						
Change in IAT	0.031	0.293	0.112	0.391*	0.501**	0.145
*PT*						
Change in IAT	−0.008	0.344	0.432*	0.26	0.507**	0.444*

IAT, Young’s Internet Addiction Test; SAS, Self Rating Anxiety Scale; SDS, Self Rating Depression Scale; Y-BOCS, Yale-Brown Obsessive-Compulsive Scale; BIS-11, Barratt Impulse Scale; PSQI, Pittsburgh Sleep Quality Index.

*P < 0.05; **P < 0.01. Bold values indicate statistical significance.

## Discussion

Recently, some studies indicated that PIU has strong association with internalizing and externalize disorders (50, 51). On the basis of a biopsychosocial model, Cerniglia thought that IAD as a result of a mutual influence of individual, psychological profile, and social environment (52). Studies have shown that depression, anxiety and impulsive are common comorbid diseases of PIU (53, 54). Simultaneously, previous studies have reported that excessive time spent online can reduce the required night sleep with internet addiction patients, furthermore leading to sleep disorders (55, 56). Our previous research also found that PIU individuals had higher SDS, SAS and BIS-11 scores, and more poor sleep quality compared with individuals without PIU (33). In present study, the correlation analysis results indicated that IAT scores of PIU individuals were positively correlated with scores of SAS, SDS, Y-BOCS and PSQI which were consistent with the results of previous studies. (57, 58). In a systematic review, Liu et al. (25) found that psychotherapy can make positive change to psychoticism, compulsive Internet use and interpersonal issues for patients with internet addiction. Moreover, some researchers reported that electro-acupuncture can improve mental symptoms in patients with Internet addiction disorder (59). In our study, both the two treatment measures could reduce the degree of Internet addiction, the symptoms of depression and anxiety, and improve sleep quality, impulsive and compulsive states in patients with PIU. Although after treatment there was no significant difference in the scores of IAT, SAS, SDS, Y-BOCS, BIS and PSQI between the two groups, the correlation analysis of the EA group showed that the change in IAT scores was positively correlated with the change in Y-BOCS and BIS scores, and the correlation analysis of the PT group showed that the change in IAT scores was positively correlated with the change in SDS, BIS and PSQI scores. Our results indicated that improvement in anxious symptoms, impulsiveness and sleep quality in PIU patients was associated with improvement in Internet addiction severity, and prompting the mechanism of the two interventions may be different.

MAOA gene is located on the short arm of the X chromosome, and some scholars found the genetic variation in MAOA effects on emotion, behavior and substance dependence. One study indicated that lower MAOA activity is associated with impulsive aggressive behavior (60). Du et al. suggested the MAO-A gene polymorphisms may be involved in the pathogenesis of major depression (61). Fite et al. study revealed that MAOA variant has association with tobacco and cannabis use (62). MAOA catalyzes the degradation of monoamine neurotransmitters, including 5-HT, DA and NE (29). In previous studies showed IAD was related to the dopamine (DA) system and serotonin (5-HT) systems, as like other substance addiction (52, 63). Therefore, MAOA is an important candidate gene for investigating PIU. Our study did not observe the MAOA levels was correlated with IAT scores, SAS scores, SDS scores, Y-BOCS scores, BIS scores and PSQI scores. Interesting after 40 days of treatment, we only found the MAOA expression of the PT group was increased, and there was no significant change in EA group. The reason may be related to the difference of treatment mechanism between the two groups. According to our previous study (19), it is speculated that electroacupuncture maybe improve symptoms by regulating reward regions of PIU patients. Furthermore, after treatment, the upregulation of MAOA gene expression in the PT group may be affected by monoamine neurotransmitters. MAOA gene activity is associated with the metabolism of monoamine neurotransmitters (32). Luo et al. (28) were summarized that platelet 5-HT level was negative related with the degree of IAD. Current research showed that psychotherapy can increase the ability of serotonin and its receptors in patients with obsessive-compulsive disorder and depression. Lissemore et al. (64) demonstrating psychotherapy can increase serotonin synthesis capacity in patients with obsessive-compulsive disorder. Karlsson et al. (65) research found that increased 5-HT1A receptor density in multiple cortical regions after psychotherapy treatment in patients with major depressive disorder. So PIU patients may cause the surge of serotonin after receiving psychotherapy, then up regulate the expression of MAOA through feedback mechanism.

So far as we know, this is the first study to examine the effect of acupuncture and psychotherapy on the mRNA levels of MAOA in PIU patients. Simultaneously, this is also the first study investigating the correlation with MAOA mRNA levels and Clinical Scale Scores in PIU patients. Although we cannot determine the difference in efficacy between EA and PT, according to our results we found that the reduction degree of internet addiction is related to the improvement of negative emotions, impulsiveness and sleep quality. In addition, EA and PT maybe has different mechanism on PIU patients. Combined with our previous research results (19), the underlying mechanisms of electroacupuncture on PIU maybe by regulating functional connectivity of reward and habit systems. While, psychotherapy may work by regulating the MAOA gene and its related neurotransmitter. To confirm our hypothesis, future studies should consider assessing a larger cohort of participants, increasing the course of treatment and follow-up time and adding the detection of neurotransmitters related to MAOA. Meanwhile, explore the correlation between these factors, so as to provide a basis for better explaining the mechanism of electroacupuncture and psychotherapy.

### Limitations

This study have limitations. Firstly, participants were recruited from college or university and the sample size of our study was relatively small that will limit the generalization of the current results. Secondly, PIU can be divided into many categories: Internet gaming disorder, Internet pornography addiction, Internet shopping addiction and so on, but we did not classify the participants. Thirdly, estimation of the follow-up effect of interventions were lacked in our research.

## Data availability statement

The original contributions presented in this study are included in the article/supplementary material, further inquiries can be directed to the corresponding author.

## Ethics statement

The studies involving human participants were reviewed and approved by Ethics Committee, Affiliated Hospital of Chengdu University of Traditional Chinese Medicine, China (NO. 2016KL-005). The patients/participants provided their written informed consent to participate in this study.

## Author contributions

TZ and YD conceptualized the study, designed the plan, and managed the project. TZ supervised the study and revised the manuscript. CZ, LZ, and CW conducted experiments. YD and CZ analyzed the data. YD wrote the first draft of the manuscript. All authors read and approved the manuscript.
